# Parenting stress and associated factors in healthcare workers after the second wave of COVID-19 in India: a two-center cross-sectional study

**DOI:** 10.3389/fpsyt.2023.1246540

**Published:** 2023-09-12

**Authors:** Vijay Kalrao, Leena Srivastava, Shruti Kumar

**Affiliations:** ^1^Department of Pediatrics, Bharati Vidyapeeth (Deemed to be University) Medical College, Pune, India; ^2^Department of Pediatrics, Shri Guru Ram Rai Institute of Medical and Health Sciences, Dehradun, India

**Keywords:** parenting stress, healthcare worker, child behavior, post-traumatic stress, anxiety, COVID-19, pandemic

## Abstract

**Background:**

High parenting stress (PS) in members of the general population during the COVID-19 pandemic was exacerbated by work-, family-, and child-related factors. However, the negative effects of PS on the mental health and work participation of healthcare workers (HCWs) have received limited attention. This study aimed to examine the proportion of severe PS among HCWs and identify its contributory factors.

**Methods:**

This cross-sectional survey was conducted in two COVID-19-care hospitals attached to medical colleges in India between November 1 and December 24, 2021, following the delta variant-driven second wave of COVID-19. The study recruited 662 HCW parent and child dyads (aged 1.5–18 years) and assessed workplace, family, and child-related characteristics. The Parenting Stress Scale (PSS) and Child Behavior Checklist (CBCL) were used to identify severe PS and child behavioral issues, respectively. Univariable and multivariable logistic regression analyzes were used to identify the significant and independent risk factors associated with severe PS, respectively.

**Results:**

Equal proportions of medical and paramedical HCWs completed the survey [mean age: 36.96 ± 5.89; female: 466 (70%)]. The median PSS score of HCWs was 33 [interquartile range (IQR): 28–39], and 23% (155/662) of the HCW parents experienced severe PS. The independent predictors of severe PS included the female sex [adjusted odds ratio (aOR): 3.31; 95% confidence interval (CI): 1.74–6.29], HCWs with >15-day postings in COVID-19 care (aOR: 3.74; 95% CI: 1.53–9.16), having children with behavioral issues (aOR: 3.49; 95% CI: 1.29–9.48), HCWs at the Dehradun center (aOR: 2.25; 95% CI: 1.24–4.10), having an HCW spouse simultaneously working in COVID-19 care (aOR: 1.88; 95% CI: 1.01–3.49), and HCWs with joint families (aOR: 1.93; 95% CI: 1.17–3.18).

**Conclusion:**

Overall, 23% of the cohort of HCWs continued to experience severe PS after the second COVID-19 wave driven by the delta variant in India. Routine screening of HCWs for PS using the PSS or similar measures, anticipatory guidance for parenting, and targeting at-risk HCWs with appropriate supportive measures may help reduce the incidence of severe PS and optimize the participation of HCWs in the fight against current and future pandemic-like situations.

## 1. Introduction

The World Health Organization has recognized that healthcare workers (HCWs) are vulnerable to physical and mental issues due to their direct or indirect contact with COVID-19 patients (1). It also highlights several knowledge gaps regarding appropriate and feasible approaches to support the physical and psychosocial well-being of HCWs providing care to COVID-19 patients. Compared with the general population, HCWs reported greater anxiety, depression, insomnia, and pandemic-related stress during the COVID-19 pandemic (2–5). HCWs who were also parents experienced greater stress than non-parents during the COVID-19 pandemic due to parenting-related challenges (6, 7).

Parenting stress (PS) is conceptually distinct from other forms of stress and arises from a mismatch between perceived parenting demands as the child’s primary caregiver and available parenting resources (8). Working parents, especially working mothers, who must balance personal life, work, domestic responsibilities, and raising children, are more susceptible to parenting-related stress than non-working parents (9, 10). The parents’ work, children’s well-being, education concerns, screen time, and family-related factors were among the top five parenting challenges identified during the COVID-19 pandemic (11, 12). Moreover, high pandemic-related stress was associated with poor parental outcomes in HCWs, leading to harsh parenting styles, behavioral issues in children, and adverse childhood experiences (ACEs) (13–17). In fact, 34.8% of parents reported PS-related increases in ACEs during the pandemic, with the highest lifetime occurrence reported for children who witnessed domestic violence and verbal and emotional abuse (13). Furthermore, the risk of child maltreatment (17), a higher incidence of harsh parenting (caning, spanking, use of harsh words, and yelling), and less relationship closeness between parents and children were observed in response to PS during the COVID-19 pandemic (18).

When both parents are HCWs (40% of healthcare workers marry each other) (11), they face additional child-and family-related challenges, such as prolonged separation from their children, working in high-risk environments, and concerns about transmitting the infection to their families. Additional work-related challenges that adversely affect the mental health of HCWs include extended shift times in intensive-care settings, which may also affect their physical health (19), exposure to the threat of COVID-19 transmission from working in close contact with ill or dying patients with high viral loads, suboptimal personal protection equipment (20, 21), a lack of adequate support in the working environment, and the absence of effective treatments for COVID-19 (21, 22).

In response to these challenges, many HCWs left their families or jobs to protect their family members from potential exposure to COVID-19 (23). Factors such as having school-aged children, having more than two children, staying away from children, and having a child with behavioral or developmental problems contributed to psychological distress and higher PS among HCWs (23–26).

Although COVID-19 infection is less severe in children, it may significantly affect their mental health (27). Children of HCWs are vulnerable due to the adverse effects of confinement and emotional instability caused by the absence of their parents, who are working long hours in hospitals or under quarantine. Consequently, unsupervised children experience separation anxiety and excessive worry. This is particularly observed in younger children, who rely on their parents for their daily needs (28). Home isolation and distance learning result in increased screen time for these children (29), and they may develop behavioral and emotional difficulties that predispose them to adverse health outcomes in adulthood (29–31). Notably, parenting becomes more challenging when a child exhibits problematic behavior (32, 33), and transactional relationships can occur in which PS and children’s behavioral problems impact one another over time (34).

Most studies conducted during the first wave of the COVID-19 pandemic have shown high PS in HCWs. However, data on PS persistence, particularly among HCWs during the second wave in India, are limited. PS has emerged as a vital target for interventions to address the adverse consequences of pandemics (13). With gaps in existing knowledge and unclear policies for HCWs and their families, identifying the extent of PS and its associated risk factors in HCWs is warranted.

Therefore, we conducted a cross-sectional survey to study the impact of COVID-19 on PS and evaluate the proportion of severe PS and its associated factors among HCWs after the delta variant-driven second wave of the COVID-19 pandemic in Pune and Dehradun, India.

## 2. Methods

### 2.1. Site selection

The second wave of COVID-19 infections, driven by the delta variant, surged in India between February and May, 2021, and placed an unprecedented burden on its healthcare system (35). The cities of Pune in Maharashtra and Dehradun in Uttarakhand were ranked among India’s top five cities in terms of deaths per million people (36–38). The high COVID-19 burden in these two cities, the presence of two large hospitals designated for COVID-19 care, and the high number of HCWs involved in COVID-19 care prompted our selection of these sites for the present study.

### 2.2. Study design and participants

A cross-sectional survey and analysis of HCWs in two tertiary COVID-19 healthcare referral hospitals attached to medical colleges in Pune, Maharashtra, and Dehradun, Uttarakhand, were conducted from November 1 to December 24, 2021. The PS, occupational profile, and familial characteristics of HCWs, as well as the behavioral characteristics of their children, were assessed.

Medical (doctors and nurses) and paramedical (multipurpose workers, medical social workers, ward coordinators, laboratory technicians, clerical and administrative staff, and security personnel) HCWs employed at the designated hospitals through the first and second waves of the COVID-19 pandemic were included in this study. Most HCW parents, except the security personnel, were employed full-time. As these were essential services during the COVID-19 pandemic, they had stable jobs and incomes. The median annual income of the doctors, nurses, and paramedical HCWs was approximately 1,440,000, 400,000, and 200,000 Indian Rupees (Rs), respectively. This was higher than the current average *per capita* income (Rs 172,000) for the rest of the Indian population.

**Figure 1:**
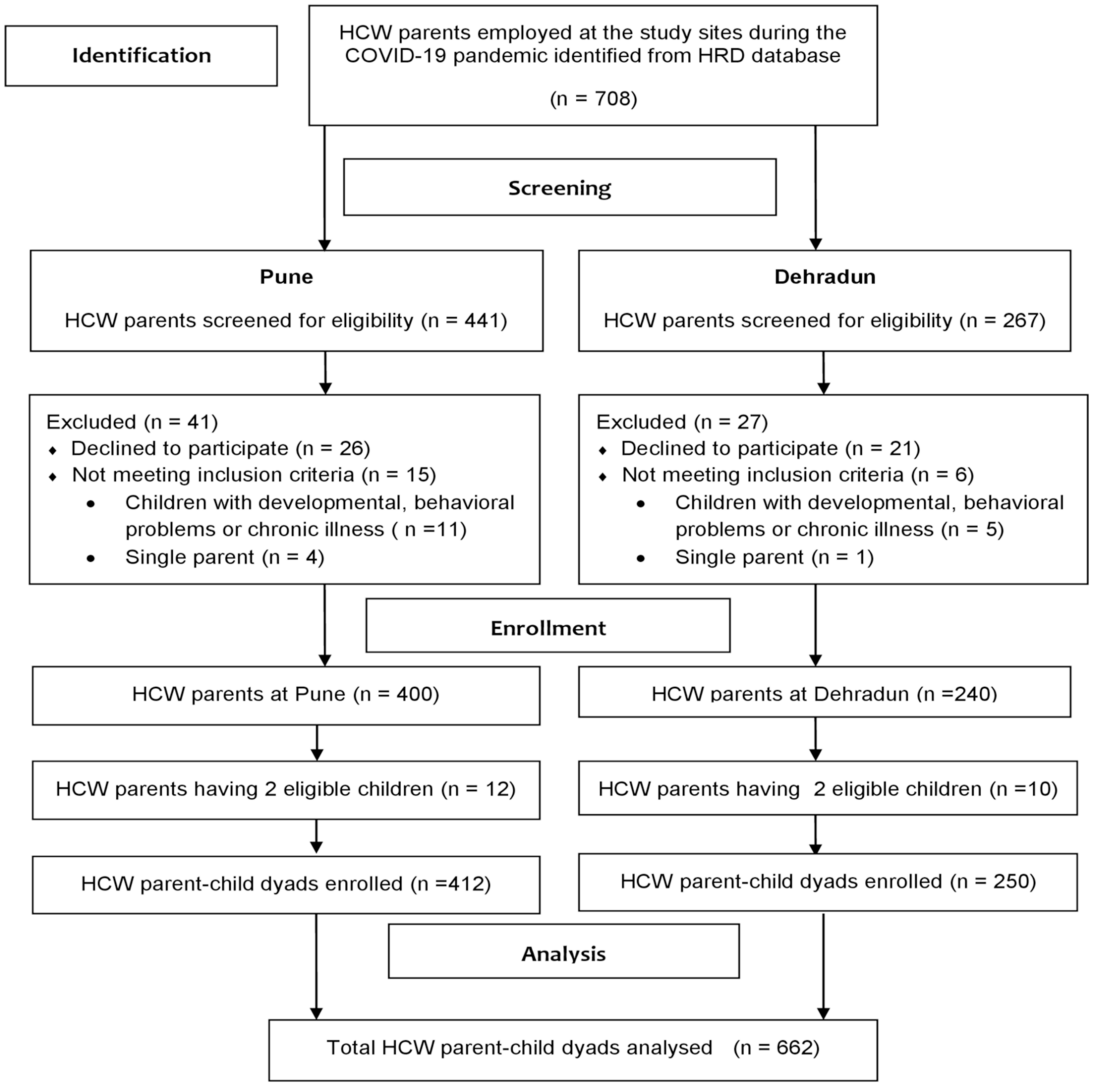
Flow diagram of HCW parent identification and enrollment in the study. HCW, healthcare worker; HRD, human resources department.

The Human Resource Development Department and HCW database at both hospitals were consulted to identify HCWs with children aged 1.5–18 years. For HCWs with two or more children, only one child from each age category (1.5–6 and 6–18 years) per family, with a maximum of two children in total, was eligible for inclusion in the study. As a result, 22 HCWs had two children belonging to different age groups. The proportion of HCWs with two-child entries was low; therefore, the data were considered to be independent. Finally, 662 HCW parent–child dyads were included in the analyzes. Figure 1 depicts the enrollment process of the study subjects. All participants provided written informed consent, and the study was reviewed and approved by the Bharati Vidyapeeth (Deemed To Be University) Medical College Institutional Ethics Committee (DCGI Reg. No. ECR 518/Inst/MH/2014/RR–17; Ref: BVDUMC/IEC/04, Dated Aug 05, 2021).

### 2.3. Measures for assessing PS and child behavior

A self-designed questionnaire captured the occupational (direct COVID-19 patient-care duration, place of work, clinical role, and work experience), COVID-19 infection (COVID-19 infection history in self, self-quarantine, or self-isolation), family (spouse work details, family type, dynamics, family history of COVID-19 infection or death, and psychosocial factors), and child-related (number of children, age, sex, and screen time) characteristics of HCWs. Additional data, such as the age and sex of HCWs, were also obtained. A trained psychologist interviewed the HCWs and assisted them in completing the Parental Stress Scale (PSS) (39) to assess their PS levels and the parent-rated Child Behavior Checklist (CBCL; CBCL 1.5–5 and CBCL 6–18 as appropriate) to assess their child’s behavioral problems (40). The parents were asked to rate their stress during the high-burden period of the second COVID-19 wave due to the delta variant (February 2021 to May 2021). Furthermore, in the parent-reported CBCL, they were asked to rate their child’s behavioral issues during the previous 2–6 months.

### 2.4. Parenting stress scale

The PSS is an instrument that assesses the positive and negative aspects of parenthood and measures PS in parents of children with typical development and those with clinically problematic behaviors (39). It contains an 18-item self-report questionnaire assessing the parent–child relationship and rates each item on a 5-point Likert scale. Certain items on the scale (items 1, 2, 5, 6, 7, 8, 17, and 18) are reverse-scored prior to computing the PSS score. The possible range of PSS scores is 18 (low stress) to 90 (high stress). The PSS has been validated in India with a good internal consistency (coefficient alpha of 0.83), a test–retest reliability of 0.81, and demonstrated satisfactory convergent validity (41). Although no absolute cutoff values for PSS scores depict the presence or absence of PS, previous studies have used mean/median scores to compare PS between groups (13, 41, 42).

### 2.5. Child behavior checklist

The CBCL is a self-administered questionnaire completed by parents/caregivers or children. It has two versions, CBCL/1.5–5 and CBCL/6–18 (40), and assesses emotional and behavioral problems in children and adolescents. A translated version has been validated for use in India to assess child and adolescent psychiatric disorders (43). For children aged 1 year and 6 months to <5 years and 6 months, we used the parent-administered CBCL/1.5–5 version, and the parent-administered CBCL/6–18 version was used for children aged ≥5 years and 6 months. Responses were assessed using a 3-point Likert scale and raw scores were used to obtain the corresponding t-scores for internalizing problems, externalizing problems, and total problem scores. The total problems score on CBCL is a valid measure for both emotional and behavioral problems. In this study, based on standard broad-scale cutoffs, children with clinical symptoms or at risk of problem behavior were considered to have behavioral problems.

### 2.6. Statistical analysis

The baseline characteristics of the HCWs are summarized using frequencies and percentages for categorical variables. PSS scores are summarized using the median and interquartile range (IQR) to denote the level of PS and were compared across groups using the Wilcoxon rank-sum test. As no standardized PSS score cutoffs are available for estimating severe PS, the PSS scores were categorized using medians with IQR cutoffs, and a score greater than the third quartile (Q3) was considered to indicate severe PS. For the CBCL, a standard cutoff t-score of ≥60 (borderline clinical risk for problem behaviors and clinical symptoms) was used to categorize the presence or absence of behavioral problems in children (40).

The potential risk factors were categorized as follows: hospital site (Dehradun or Pune), work profile of the HCW parent [doctor, nurse, multipurpose worker (MPW), or other HCW], medical HCW (yes or no), sex of the HCW (male or female), age category (<30, 30–40, or > 40 years), COVID section work (yes or no) and duration (<15 or ≥ 15 days), personal COVID infection test status (positive or negative), quarantined and stayed away from children (yes or no) and duration (<15 or ≥ 15 days), HCW spouse simultaneously working in COVID care (yes or no), family type (joint or nuclear), family history of COVID infection or death (yes or no), and the number of children aged ≤18 years (1 or ≥ 2), their sex (male or female), screen time (≥5 or < 5 h), and behavioral problem status (yes or no). Univariable logistic regression was used to estimate odds with a 95% confidence interval (CI) to identify the risk factors associated with severe PS. Factors associated with severe PS with a *p*-value <0.1 in the univariable analysis were used in the multivariable model to estimate the adjusted odds ratio (aOR) with a corresponding 95% CI to identify independent risk factors for severe PS. SPSS version 28.0 (IBM Corp., Armonk, NY, United States) was used for all analyzes, and statistical significance was set at *p* < 0.05.

## 3. Results

### 3.1. Baseline characteristics of the HCWs

Table 1 summarizes the frequencies and percentages of the work, family, and child-related variables of the HCWs. The entire study sample included 662 HCW parent–child dyads, with 250 and 412 from Dehradun and Pune, respectively. Notably, medical (334 doctors and nurses) and paramedical HCWs (328 MPWs and other HCWs) were equally represented. A total of 466 (70%) HCWs were female. Being a COVID-care facility, the majority of HCWs [531 (80%)] interacted directly with patients with COVID-19. In addition, 178 (27%) HCWs had spouses working simultaneously in COVID-19 care at other study sites or care facilities.

**Table 1:** Baseline characteristics of the HCWs.

Variables	Dehradun (*n* = 250) *n* (%)	Pune (*n* = 412) *n* (%)	Total (*n* = 662) *n* (%)
Age of the children			
1.5 to 5 years of age	78 (31%)	104 (25%)	182 (27%)
6 to 18 years of age	172 (69%)	308 (75%)	480 (73%)
HCW characteristics			
**HCW category**			
Doctor	62 (25%)	111 (27%)	173 (26%)
Nurse	61 (24%)	100 (24%)	161 (24%)
MPW	40 (16%)	88 (21%)	128 (19%)
Other HCWs	87 (35%)	113 (27%)	200 (30%)
**Sub-category**			
Medical HCWs (doctors/nurses)	123 (49%)	211 (51%)	334 (50%)
Paramedical HCWs (MPW/Other)	127 (51%)	201 (48%)	328 (50%)
**Doctor’s specialization**			
Physician/intensivist	25 (41%)	58 (52%)	83 (48%)
OBGYN	6 (10%)	6 (5%)	12 (7%)
Preclinical/paraclinical	8 (13%)	18 (16%)	26 (15%)
Surgeon	23 (36%)	29 (26%)	52 (30%)
**Age category of the HCW (years)**			
<30	28 (11%)	68 (17%)	96 (15%)
30–40	153 (61%)	235 (57%)	388 (59%)
>40–50	63 (25%)	104 (25%)	167 (25%)
>50	6 (2%)	5 (1%)	11 (2%)
**Sex of the HCW**			
Female	151 (60%)	315 (76%)	466 (70%)
Male	99 (40%)	97 (24%)	196 (30%)
**Work experience in hospital**			
<5 years	59 (24%)	191 (46%)	250 (38%)
≥5 years	191 (76%)	221 (54%)	412 (62%)
**Place of work**			
ICU (COVID)	27 (11%)	204 (51%)	231 (35%)
Indoor (COVID)	107 (43%)	96 (24%)	203 (31%)
OPD (COVID)	56 (22%)	41 (10%)	97 (15%)
Indoor (non-COVID)	8 (3%)	7 (2%)	15 (2%)
OPD (non-COVID)	27 (11%)	51 (12%)	78 (12%)
Laboratory (non-COVID)	25 (10%)	13 (3%)	38 (6%)
**Overall**			
COVID-section work	190 (76%)	341 (83%)	531 (80%)
Non-COVID-section work	60 (24%)	71 (17%)	131 (20%)
**Duration of COVID-care work**			
<15 days	38 (20%)	67 (20%)	105 (20%)
≥15 days	152 (80%)	274 (80%)	426 (80%)
**COVID test results in the HCWs**			
COVID-negative	130 (52%)	253 (61%)	383 (58%)
COVID-positive	120 (48%)	159 (39%)	279 (42%)
**Quarantine and stay away from children**			
Yes	122 (49%)	144 (35%)	266 (40%)
No	128 (51%)	268 (65%)	396 (60%)
**HCW spouse simultaneously working in COVID-care**			
Yes	89 (36%)	88 (21%)	177 (27%)
No	161 (64%)	324 (79%)	485 (73%)
**One parent with the child at all times**			
Yes	178 (71%)	259 (63%)	437 (66%)
No	72 (29%)	153 (37%)	225 (34%)
Family characteristics			
**Type of family**			
Joint	116 (46%)	176 (43%)	292 (44%)
Nuclear	134 (54%)	236 (57%)	370 (56%)
**Worried about infection in family**			
Yes	235 (94%)	338 (82%)	573 (87%)
No	15 (6%)	74 (18%)	89 (13%)
**COVID infection in family**			
Yes	73 (29%)	154 (37%)	227 (34%)
No	177 (71%)	258 (63%)	435 (66%)
**COVID death in family**			
Yes	6 (2%)	41 (10%)	47 (7%)
No	244 (98%)	371 (90%)	615 (93%)
Child’s characteristics			
**Number of children aged ≤18 years**			
1	129 (52%)	200 (49%)	329 (50%)
≥2	121 (48%)	212 (51%)	333 (50%)
**Sex of the child**			
Female	113 (45%)	193 (47%)	306 (46%)
Male	136 (55%)	220 (53%)	356 (54%)
**Child’s screen time**			
<5 h	187 (75%)	279 (69%)	466 (71%)
≥5 h	63 (25%)	133 (31%)	196 (29%)
**Child’s COVID status**			
Positive	8 (3%)	36 (9%)	44 (7%)
Negative	242 (97%)	376 (91%)	618 (93%)
**Total behavioral problem**			
Yes	10 (4%)	25 (6%)	35 (5%)
No	240 (96%)	387 (94%)	627 (95%)

HCW, healthcare worker; MPW, multipurpose worker; OBGYN, obstetrician and gynecologist; ICU, intensive care unit; OPD, outpatient department.

### 3.2. Comparison of the median PSS scores in HCWs across categories

PSS scores were summarized using median and IQR values and compared across groups using a Wilcoxon rank-sum test (Table 2). The overall median PSS score among the 662 HCW parents was 33 (IQR: 28–39). Several factors were associated with significantly higher median PSS scores (*p* value <0.05), including HCWs from the Dehradun site, doctors, HCWs older than 50 years, working in non-COVID-19 areas, HCWs with a history of testing positive for COVID-19, HCWs quarantined away from their children, HCWs with a spouse simultaneously working in COVID care, neither of the HCW parents present with the child, and HCWs with children exhibiting problem behaviors.

**Table 2:** Comparison of the median PSS scores across categories.

	Children aged 1.5–18 years	
	Median PSS score (IQR)	*P*-value^@^
Overall PSS score	33 (28–39)	-
Site-specific		
Pune	30 (26–37)	<0.001*
Dehradun	36 (31–41)	
HCW characteristics		
HCW category		
Doctor	34 (30–41)	<0.001*
Nurse	31 (27–38)	
MPW	32 (26–39)	
Others	33 (29–39)	
Sub-category		
Medical HCWs (doctors/nurses)	33 (28–39)	0.56
Paramedical HCWs (MPW/Others)	33 (27–39)	
Doctor’s specialization		
OBGYN	34 (29–41)	0.67
Preclinical/paraclinical	35 (27–41)	
Physician/intensivist	35 (31–41)	
Surgeon	33 (29–41)	
Age category of HCW (years)		
<30	31 (25–38)	0.03*
30–40	33 (28–40)	
41–50	31 (27–38)	
>50	33 (29–40)	
Sex of HCW		
Male	32 (28–37)	0.12
Female	33 (28–40)	
Work experience in hospital		
<5 years	33 (27–39)	0.51
≥5 years	33 (28–39)	
Place of work		
ICU (COVID)	30 (26–36)	<0.001*
Indoor (COVID)	33 (28–38)	
OPD (COVID)	34 (29–39)	
Indoor (non-COVID)	35 (30–41)	
OPD (non-COVID)	36 (31–40)	
Laboratory (non-COVID)	39 (31–44)	
Place of work		
Non-COVID	36 (30–41)	<0.001*
COVID	31 (27–38)	
Duration of COVID-care work		
<15 days	32 (27–37)	0.95
≥15 days	31 (27–39)	
COVID test results in the HCWs		
Negative	31 (27–38)	<0.001*
Positive	35 (29–41)	
Quarantine and stay away from children		
Yes	34 (29–40)	<0.01*
No	32 (27–38)	
HCW spouse simultaneously working in COVID-care		
Yes	35 (30–41)	<0.001*
No	31 (27–38)	
One parent with the child at all times		
Yes	32 (27–39)	0.01*
No	34 (29–39)	
Family characteristics		
Type of family		
Nuclear	32 (27–38)	0.06
Joint	34 (28–40)	
Family h/o COVID-positive infection		
Yes	34 (29–40)	0.05
No	32 (27–39)	
COVID-related death in family		
Yes	31 (27–36)	0.09
No	33 (28–39)	
Child characteristics		
Age of the child		
1.5–5 years	33 (28–40)	0.3
6–18 years	33 (27–39)	
Number of children aged ≤18 years		
1	33 (28–39)	0.2
≥2	31 (27–39)	
Sex of child		
Male	32 (27–39)	0.12
Female	33 (29–39)	
Child’s COVID status		
Positive	31 (27–41)	0.6
Negative	33 (28–39)	
Screen time		
<5 h	33 (27–39)	0.74
≥5 h	32 (29–38)	
Total behavioral problem		
Yes	39 (36–44)	<0.001*
No	32 (27–39)	

HCW, healthcare worker; OBGYN, obstetrics and gynecology; ICU, intensive care unit; OPD, outpatient department; PSS, Parental Stress Scale; IQR: interquartile range; h/o, history of; ^@^Wilcoxon rank-sum test; *statistically significant values (*p* < 0.05).

### 3.3. Factors associated with severe PS in HCWs

We considered PSS scores higher than the third quartile (PSS > Q3, 39) as severe PS. Consequently, 155/662 (23%) HCWs experienced severe PS. Univariable logistic regression was used to estimate the OR with corresponding 95% CIs and identify risk factors for severe PS (Table 3). Living in Dehradun [OR: 2.61, 95% CI (1.81–3.76)], working as a doctor [OR: 1.88, 95% CI (1.12–3.13)], working in a non-COVID-19 section [OR: 2.34, 95% CI (1.61–3.41)], working longer than 15 days in COVID-19 care [OR: 2.50, 95% CI (1.16–5.39)], having a history of COVID-19 infection [OR: 1.91, 95% CI (1.33–2.75)], having a spouse working simultaneously in COVID-19 care [OR: 2.02, 95% CI (1.38–2.97)], being quarantined away from children (OR: 1.45, 95% CI [1.01–2.09]), and having children with behavioral problems [OR: 3.08, 95% CI (1.54–5.94)] were significant risk factors for severe PS.

**Table 3:** Factors associated with severe parenting stress in HCWs.

					Univariable analysis^@^		Multivariable analysis^#^	
Variable		PSS score		Total	OR (95% CI)	*P*-value	aOR (95% CI)	*P*-value
		>Q3	≤Q3					
Hospital site	Dehradun	86	164	250	2.61 (1.81–3.76)	<0.001*	2.25 (1.24–4.10)	0.01*
	Pune	69	343	412	1			
HCW Type	Doctor	52	121	173	1.88 (1.12–3.13)	0.02*	2.14 (0.94–4.85)	0.07
	MPW	27	101	128	1.17 (0.65–2.08)	0.60	1.17 (0.77–3.78)	0.19
	Other	46	154	200	1.30 (0.77–2.18)	0.31	2.12 (0.97–4.63)	0.06
	Nurse	30	131	161	1		1	
Medical HCW	Yes	82	252	334	1.14 (0.79–1.63)	0.49		
	No	73	255	328	1			
Doctor type	OBGYN	3	9	12	0.77 (0.19–3.10)	0.72		
	Preclinical/ paraclinical	9	17	26	1.23 (0.48–3.13)	0.67		
	Surgeon	14	37	51	0.88 (0.41–1.90)	0.74		
	Physician/intensivist	25	58	83	1			
Age of HCW (years)	30–40	101	287	388	1.52 (0.87–2.67)	0.14		
	>40	36	142	178	1.10 (0.58–2.06)	0.77		
	<30	18	78	96	1			
Sex of HCW	Female	119	347	466	1.54 (1.02–2.34)	0.04*	3.31 (1.74–6.29)	<0.001*
	Male	36	160	196	1		1	
Family type	Joint	78	214	292	1.39 (0.97–1.98)	0.08	1.93 (1.17–3.18)	0.01*
	Nuclear	77	293	370	1		1	
Years of experience	≥5	100	311	411	1.14 (0.78–1.66)	0.49		
	<5	55	195	250	1			
Work in the COVID section	No	75	145	220	2.34 (1.61–3.41)	<0.001*	1.77 (0.88–3.52)	0.11
	Yes	78	353	431	1		1	
Duration of COVID-care work	>15 days	91	314	405	2.50 (1.16–5.39)	0.02*	3.74 (1.53–9.16)	<0.001*
	<15 days	8	69	77	1		1	
COVID test results in the HCWs	Positive	84	195	279	1.91 (1.33–2.75)	0.001*	1.68 (0.94–3.00)	0.08
	Negative	70	310	380	1		1	
One parent with children all times	Yes	99	338	437	0.88 (0.61–1.29)	0.52		
	No	56	169	225	1			
HCW spouse simultaneously working in COVID-care	Yes	59	118	177	2.02 (1.38–2.97)	<0.001*	1.88 (1.01–3.49)	0.04*
	No	96	388	484	1		1	
Quarantined and stayed away from children	Yes	73	191	264	1.45 (1.01–2.09)	0.04*	1.43 (0.79–2.56)	0.24
	No	82	312	394	1		1	
Quarantine duration	≥15 days	49	111	160	1.55 (0.88–2.75)	0.13		
	<15 days	23	81	104	1			
Worried about family infection	Yes	139	434	573	1.46 (0.82–2.59)	0.20		
	No	16	73	89	1			
COVID infection in the family	Yes	62	165	227	1.38 (0.95–2.0)	0.09		
	No	93	342	435	1			
Death in the family due to COVID	Yes	7	40	47	0.55 (0.24–1.26)	0.16		
	No	148	467	615	1			
Number of children aged ≤18 years	≥2	73	260	333	0.85 (0.59–1.21)	0.36		
	1	82	247	329	1			
Child’s screen time	≥5 h	43	148	191	0.92 (0.62–1.37)	0.68		
	<5 h	112	354	466	1			
Child’s COVID-positive status	Yes	12	32	44	1.25 (0.63–2.48)	0.53		
	No	143	475	618	1			
Total behavioral problem	Yes	17	20	37	3.08 (1.54–5.94)	0.001*	3.49 (1.29–9.48)	0.01*
	No	137	488	625	1		1	

HCW, healthcare worker; MPW, multipurpose worker; OBGYN, obstetrician and gynecologist; PSS, Parental Stress Scale; Q3, third quartile median score; OR, odds ratio; aOR, adjusted odds ratio; CI, confidence interval. ^@^Univariable logistic regression. ^#^Multivariable logistic regression. *Statistically significant values (*p* < 0.05).

The variables found to be statistically significant in the univariable analysis (*p* < 0.05) and other variables with *p*-values <0.10 were selected for multivariable analysis wherein the aOR was estimated, and independent risk factors for severe PS were identified (Table 3). It was observed that HCWs from Dehradun [aOR: 2.25, 95% CI (1.24–4.10)] with spouses working simultaneously in COVID-19 care [aOR: 1.88, 95% CI (1.01–3.49)] and those from joint families [aOR: 1.93, 95% CI (1.17–3.18)] were twice as likely to experience severe PS. Furthermore, female HCWs [aOR: 3.31, 95% CI (1.74–6.29)], those working longer in COVID-19 care [aOR: 3.74, 95% CI (1.53–9.16)], and HCWs with children with behavioral problems [aOR: 3.49, 95% CI (1.29–9.48)] had more than three times the odds of developing severe PS. Notably, upon multivariable analysis, doctors, HCWs working in non-COVID-19 areas, those with a history of COVID-19 infection, or those quarantined away from their children were not independently associated with severe PS.

## 4. Discussion

The second wave of the COVID-19 pandemic caused by the delta variant waned by June 2021 in India. In the current study, a cross-sectional survey assessed PS in HCWs and its contributory factors during the period spanning from November 1 to December 24, 2021. The study included 662 HCW parent–child dyads, comprising medical and paramedical HCWs, from tertiary-level COVID-19 care facilities in medical college hospitals in two different geographic regions of India. Although higher PSS scores indicate greater PS, no absolute PSS cutoff scores differentiate the level of PS. A translated version of the PSS in Hindi was validated and used for Indian parents of children with and without a clinical diagnosis, using cutoff scores of ≥45 to estimate PS (42). For the analyzes in the current study, a PSS score greater than the third quartile (>39) was considered to indicate severe PS.

### 4.1. PS in HCWs

Few studies have focused on PS in the general population during the COVID-19 pandemic. The general and perceived stress during the COVID-19 pandemic correlated well with PS (41, 44). Although data on PS in HCWs are limited, clinically higher PS in HCWs than in non-HCWs has been indicated (17, 24, 45, 46). Conversely, some recent reports have observed significantly fewer mental health problems among HCWs than among other essential workers and the general population. Better mental health in HCWs may translate to similar or reduced PS in HCW parents during the pandemic compared with the general public (47–49).

In the current study, HCW parents had a lower median PS score of 33 (IQR: 28–39) compared with data from population studies in Indian non-HCW parents prior to the COVID-19 pandemic (mean PS score: 36.68 ± 6.222), during the COVID-19 pandemic (mean PS score: 36.86 ± 8.24) (41, 42), and in a survey of German non-HCW parents during the highest COVID-19 burden periods (mean PS score: 36.93 ± 10.45) (13). Notably, approximately one-quarter of HCWs in the present study experienced severe PS.

The novelty of the pandemic, its widespread effects, limited resources, and anxiety concerning infection and transmission to families significantly impacted the mental and physical health of HCWs during the initial stages of the pandemic (49–51). The overall lower median PS scores in HCWs compared with the general population after the second COVID-19 wave observed in the current study could be due to the timing of the study, as psychological distress in HCWs decreased during the later phases of the pandemic (52).

Furthermore, both COVID-19 waves in India occurred later than they did in Western countries (53). Therefore, the knowledge gained from experience may have helped health managers and HCWs respond more efficiently, become pandemic-ready, and ease COVID-19-related anxiety (54). The perception of control over stressful events in HCWs was found to help them cope with and adapt to stressful situations, as well as lower their overall stress. However, despite the perceived improvement regarding the impact of COVID-19 among HCWs, PS continues to be high among other parents who still feel vulnerable (44).

### 4.2. Occupational characteristics and severe PS in HCWs

Importantly, PS is also associated with employment (39). The prevalence of anxiety, pandemic stress, and PS in a particular HCW category may be related to their demographic distribution in a given setting, available support, perceived protection, share of the workload, time spent with infected patients, and information and training received (51).

In this study, parents from the COVID-19 care facility at Dehradun had more than double the odds of severe PS than those at the Pune site. Although both study sites were medical college hospitals with better facilities than most other health centers in India, there was a difference in the reported levels of severe PS between the HCWs from these two centers. This relates to differences in the standards of healthcare facilities, health infrastructure, and preparedness. Moreover, site-specific healthcare steps and the different competencies and strategies adopted to handle an epidemic may be contributing factors. HCWs working in other healthcare settings throughout India, primarily in rural and underdeveloped regions, have minimal resources and are not optimally prepared to handle the COVID-19 pandemic; therefore, they may be affected to a greater extent.

In this study, doctors had significantly higher odds of having severe PS than nurses. However, this difference was not significant after adjusting for confounding factors. Notably, medical and paramedical HCWs had similar odds of developing severe PS. Previous studies comparing the psychological impact of the COVID-19 pandemic on HCWs found that paramedical HCWs experienced more fear, anxiety, and depression than medical HCWs (55). Reduced access to proper psychological support, minimal first-hand medical information on the outbreak, and a lack of intensive training, personal protective equipment, and infection control measures may lead to pandemic-related stress and, thus, higher PS among paramedical HCWs (55).

By contrast, studies conducted in the early phase of the COVID-19 pandemic found severe psychological problems in medical HCWs (56, 57) compared with paramedical HCWs, due to their proximity to infectious patients. However, the parent–child relationship, measured by parent–child interaction dysfunction, was most affected in paramedical HCWs (24). An early-phase study investigating psychological distress among HCWs from the Asia-Pacific region using a machine-learning approach found that nurses had the lowest stress levels compared with other HCWs (58). Additionally, frontline nurses generally reported lower stress levels than other nurses and the general population (44).

Many HCWs (approximately 40%), especially doctors, marry other HCWs and may provide hospital-based care at the same time as their spouses (11). Thus, the childcare crisis is exacerbated by the absence of HCW parents from the home (59). In this study, it was observed that HCWs with spouses working simultaneously in COVID-19 care were independently associated with twice the odds of severe PS. Moreover, two-thirds (66%) of doctors, compared with one-third (27%) of all HCWs at our study sites, had spouses who were HCWs and simultaneously involved in providing care in the same or another COVID-19 care setting. Notably, doctors at our study sites had to share a greater responsibility for decision-making. The dynamic work environment, dealing with dying patients, and revealing bad news to relatives may increase anxiety and pandemic-associated stress (60). These factors may account for the higher levels of PS among doctors than nurses. If both spouses are engaged in COVID-19 care, remedial steps such as deploying only one to COVID-19 care at a time, allowing flexible work hours or self-scheduling to improve work-life balance, and extending rest days may mitigate the childcare crisis and help reduce PS.

### 4.3. Work-setting and severe PS in HCWs

HCWs in high-risk COVID-19 care areas are more likely to report higher PS due to a higher risk of contracting the infection than those working in non-COVID-19 settings (51, 61). However, in the current study, HCWs working in designated non-COVID areas had higher PS than those working in areas designated for the care of COVID-19 patients [COVID indoor wards, intensive care units, and outpatient departments (OPDs)]. Upon univariable analysis, the odds of having severe PS were almost double among HCWs working in the designated non-COVID-care areas compared with those working in the COVID-19-care zones. However, the difference in PS was not statistically significant after adjusting for the other variables. In an earlier study, medical staff working in respiratory, emergency, infectious disease, and ICU departments were in close contact with infected patients (56) and were greatly concerned regarding infection (61). They faced higher workloads and higher levels of exhaustion, depression, anxiety, and post-traumatic stress than HCWs caring for non-COVID-19 patients in other units (44, 51, 62, 63).

The contrasting results from this study may be related to the timeframe and location of maximum stress. Most other studies were conducted in the early phases of the pandemic and found higher stress among HCWs working in COVID-19 care areas due to the uncertainty surrounding the pandemic being at its peak (57). At this time, HCWs in acute medical units and those working closely with patients in COVID-19 care areas were at the highest risk of infection (62). In the later phases of the pandemic, better preparedness helped reduce infection rates in these areas, and threat perception waned to a certain extent (64). Similarly, another study found that the severity of vicarious traumatization in non-frontline nurses and the general public was significantly higher than that in frontline nurses who came in close contact with patients with COVID-19 (65). Feelings of self-efficacy and control come from competency, adequate medical resources, and education regarding preventive and basic standard measures (66). These individuals feel protected, leading to positive coping strategies in response to stress, assisting in the mitigation of PS (44, 67). High-risk areas such as COVID-19 care areas received most of the resources (68), and this targeted approach indicated the elevated threat perception and anxiety of workplace-related exposure to COVID-19 in these areas remained high among HCWs working in non-COVID-19 areas (55). Therefore, lower self-efficacy and control, coupled with higher perceived stress, may account for the higher PS in HCWs working in low-risk “non-COVID-19” areas in our study.

### 4.4. Severe PS and the sex of the HCWs

Notably, being a female HCW was an independent risk factor for severe PS, with more than three times the odds of experiencing severe PS than their male counterparts. By contrast, previous studies reported no differences in PS between fathers and mothers of typically developing children in the general population or HCWs, both during the pre-pandemic period (39) and during previous epidemics or recently published studies during the COVID-19 pandemic (13, 62, 69). However, employed mothers are multi-tasked and experience more stress because they are more likely to bear the burden of the pandemic due to their higher domestic care and work responsibilities (70, 71). Culturally, in most societies, including India, mothers share the primary burden of childcare and household chores (72) and have reported higher PS during the COVID-19 pandemic (41). Studies conducted in the earlier phase of the pandemic also reported higher PS and parental burnout, as well as lower well-being, in females than in their male counterparts (2, 24, 46, 73–76). In our study conducted during the later stages of the COVID-19 pandemic, the vulnerability of Indian female HCWs to severe PS persisted.

### 4.5. COVID-19 infection-related risk and severe PS

Prolonged contact with the source of infection, higher caseloads, longer working hours, and exhaustion add to COVID-19-related anxiety (49). In our study, HCWs with more than 15 days of COVID-19 work had four times higher odds of severe PS than those with less than 15 days of COVID-19 work. HCWs, especially those on the frontline, experienced a tenfold higher risk of contracting COVID-19 (77). Owing to the nature of their work, 10–20% of all patients diagnosed with COVID-19 are HCWs (78). In addition, most COVID-19 tests were performed only in symptomatic HCWs, and therefore the COVID-19 infection rates in HCWs may be underestimated (77). In the present study, 42% of HCWs at the two study centers previously had COVID-19 infection. Coronavirus-related anxiety and a history of COVID-19 infection in HCWs are known to increase the chances of PS (24). Notably, the odds of severe PS were high in HCWs with COVID-19 infection; however, this was not significant after adjusting for confounders. Other studies on the general population and HCWs have found that having an acquaintance or family member with COVID-19 is associated with higher levels of stress, anxiety, depression, and parental distress (46, 79). However, this study did not find an association between PS in HCWs and COVID-19 infection or death in their families.

### 4.6. Family structure and severe PS in HCWs

Parents with joint families were an independent risk factor for severe PS, with twice the odds of severe PS compared with HCWs from nuclear families. For most working parents, protecting their family members from the risk of COVID-19 remains a high priority (80). In addition to the fear of infecting family members, research has shown that parental perceptions of COVID-19 are associated with increased anxiety and PS (18, 56). HCWs leave their families or jobs for extended periods due to this fear (23, 44). Parents from joint families have a support system that can benefit working parents and result in lower PS (81, 82). By contrast, some studies have reported no such association (10, 83). However, a study conducted during the current COVID-19 pandemic found higher PS in those with extended families than in those with nuclear families, similar to the findings of the present study (84). During this pandemic, the higher stress related to financial strain and risks of COVID-19 infections in the family due to the inability to quarantine in smaller houses may outweigh the beneficial effects of an extended family. This may explain the higher PS among HCWs from joint families in the present study.

### 4.7. Characteristics of children and severe PS in HCWs

The number of children, age, sex, COVID-19 positivity status, and higher screen time were not associated with severe PS in HCWs. In another India-based study during COVID-19, PS did not vary with the number of children but was higher in parents reporting more screen time in their children (41). Moreover, HCW parents of younger or school-aged children showed higher PS because parents of younger children may be more anxious about COVID-19. Younger children depend entirely on their caregivers for their daily needs. Therefore, leaving dependent children unattended for long periods of time contributes to a higher PS for such parents (2, 24, 74, 80). In parents of children aged 5–17 years, PS did not vary between the age groups but rather correlated with the number of children at home, possibly due to the higher financial burden and increased teaching responsibilities of these parents (24, 76). In this study, HCWs with children exhibiting behavioral problems experienced three times higher odds of severe PS than those with children without problem behaviors. Negative child behaviors increase the PS levels of HCWs (85), whereas children’s well-being is a protective factor against PS. Thus, the parents of children with better psychological adjustment experience fewer difficulties in their parental roles (74). Disruption of the daily routine, prolonged screen time, lack of socialization and peer contact, fear of acquiring COVID-19, and the stress of exams are contributing factors to the rise of emotional and behavioral concerns in children (80).

Previous studies have shown that PS and increased child behavioral problems correlate well in typically developing pre-school children (32, 33). Moreover, having a child with emotional problems during the pandemic was an independent risk factor for PS (46). In another India-based study, PS was higher in parents of children with developmental or behavioral problems during the course period than in typically developing children (41). Furthermore, a child’s behavioral problems and PS have a transactional relationship as having children with problem behaviors increases PS over time, while high PS can exacerbate behavioral problems in children (86).

### 4.8. Strengths and limitations of the study

Our study had several strengths. First, previous studies used online questionnaires that required participants to possess the necessary skills to operate their smartphones, computers, or tablets. However, the current study involved direct interviews with parents conducted by trained psychologists who assisted in administering the scales. This helped reduce errors due to misinterpretation of the questionnaire items. Second, the sample size was significant compared with that of previous studies, representing all categories of HCWs, including medical and paramedical, as well as mothers and fathers, unlike previous studies that focused on specific categories or sexes. Thus, our results may be more representative of PS among HCWs than those of previous studies. Third, this study had no missing data. Therefore, a bias in the interpretation of the results is unlikely.

However, this study also had some limitations. First, as this was a cross-sectional study, we can only report an association, not causation. Second, considering only one child per family in each age group may have resulted in a sampling error. However, this helped to eliminate any bias from the repetitive compilation of the same questionnaires among siblings from the same age category. Third, recall bias may have affected the interpretation of results due to the 6 months from the end of the second wave of COVID-19 to the survey. Therefore, PS in HCWs may have been underestimated, as it may have been higher during the peak of the second wave than during the survey period. Most studies on PS among HCWs use different assessment tools, and very few studies have used the PSS, which we used in our study. This leads to the fourth limitation of our study, namely that there are no standard cutoff scores when using the PSS to judge the severity of PS. We considered severe PS among HCWs as a PSS score higher than the third quartile. Although this method has not been validated in previous studies, it is more likely to be representative of severe PS in HCWs because the third quartile scores in HCWs were much higher than the mean PSS scores from previous studies among non-HCWs both during and before the pandemic. Therefore, replicability is required in future studies. Moreover, the CBCL is parent-reported and not child-reported; therefore, the underreporting of problem behaviors in children in this study was possible. Lastly, the prevalence of PS and its contributing factors may be country-or region-specific. In India, access to healthcare and other resources varies geographically and is unequal. The current study was conducted at two tertiary-level medical care college hospitals with the best infrastructure and human resources to deal with the pandemic. Notably, there was still a significant difference in the odds of severe PS among HCWs between the sites. HCW parents across other resource-starved settings in our country or elsewhere, including areas with rural healthcare, may be more affected, and the results of this study may underrepresent the overall impact of COVID-19 on the PS of HCWs. Therefore, the observations from this study may not be generalizable to other populations, healthcare facilities, or locations. However, general measures suggested to reduce PS among HCWs may still benefit all HCWs.

## 5. Conclusion

The COVID-19 pandemic, social distancing measures, and workplace pressures have exacerbated PS among HCWs who are also parents. Adequate organizational support, flexible work hours, and adequate rest periods are essential considerations for HCWs to balance childcare and job responsibilities. Moreover, ensuring self-protection through adequate infection control measures and PPE for medical and paramedical HCWs working directly or indirectly with infected patients within the same facility is crucial. Allowing self-scheduling of duties and providing rest periods for one parent at a time can also help balance childcare needs when both spouses are involved in COVID-19 care. Furthermore, anticipatory guidance that routinely screens HCWs for parenting stress and children for problem behaviors and promptly applies remedial measures may reduce PS among HCWs. Although HCWs are concerned about infecting their families, providing quarantine facilities at home may be challenging due to space constraints. Therefore, future studies should focus on the long-term consequences of severe PS on the physical and mental health of HCWs and their children and family. Healthcare systems should prioritize support for at-risk HCWs to retain their support in the fight against COVID-19. The study’s findings may help healthcare officials and government agencies formulate policies to ensure the physical and mental well-being of HCWs in future COVID-19-like pandemics.

## Data availability statement

The raw data supporting the conclusions of this article will be made available by the authors, without undue reservation.

## Ethics statement

The studies involving humans were approved by Bharati Vidyapeeth (Deemed To Be University) Medical College Institutional Ethics Committee (DCGI Reg. No. ECR 518/Inst/MH/2014/RR–17; Ref: BVDUMC/IEC/04, Dated Aug 05, 2021). The studies were conducted in accordance with the local legislation and institutional requirements. The participants provided their written informed consent to participate in this study.

## Author contributions

VK and LS conceptualized the idea and research questions and drafted the methodology and wrote the first draft of the manuscript. VK, LS, and SK contributed to the data collection, analysis, and literature review. All authors contributed to the article and approved the submitted version.

## Funding

The Central Research and Publication Unit (CRPU) of Bharati Vidyapeeth (Deemed to be University) Medical College, Pune, funded the study (ref: BVDU/MC/2249/21–22, dated 22/09/2021). The funders had no role in the study design, data collection, analysis, publication decisions, or manuscript preparation.

## Conflict of interest

The authors declare that the research was conducted in the absence of any commercial or financial relationships that could be construed as a potential conflict of interest.

## Publisher’s note

All claims expressed in this article are solely those of the authors and do not necessarily represent those of their affiliated organizations, or those of the publisher, the editors and the reviewers. Any product that may be evaluated in this article, or claim that may be made by its manufacturer, is not guaranteed or endorsed by the publisher.
